# Diet and Stress Impair Ovarian Function in Mid-life, Increasing Risk of Chronic Diseases of Aging in Primates

**DOI:** 10.1093/geroni/igab046.2552

**Published:** 2021-12-17

**Authors:** Brett Frye, Suzanne Craft, Thomas Register, Susan Appt, Mara Vitolins, Beth Uberseder, Marnie Silverstein-Metzler, Carol Shively

**Affiliations:** Wake Forest School of Medicine, Winston Salem, North Carolina, United States

## Abstract

Ovarian dysfunction increases risk for chronic diseases of aging including cardiovascular disease, depression, cognitive impairment, and bone and muscle loss which promote frailty. Psychosocial stress disrupts ovarian function and recent observations suggest that Western diet may also. Determination of causal relationships among diet, psychosocial stress, and ovarian physiology is difficult in humans. Nonhuman primates provide relevant opportunities to investigate diet and psychosocial effects on ovarian physiology and aging because, like humans, they have monthly menstrual cycles and recapitulate many aging-related processes similar to humans. We examined ovarian function in 38 socially housed, middle-aged females fed either a Western or Mediterranean diet for 26 months (~ an 8-year period for humans). During the last 12 months, we examined cycle length, peak progesterone per cycle, and frequency of anovulatory cycles using blood sampling (3/week) and vaginal swabbing (6/week). Repeated measures analysis revealed that like middle-aged women, cycle length increased, and progesterone levels fell over time, suggesting that ovarian dysfunction generally increased in our sample with time. In addition, both Western diet and the stress of low social status reduced progesterone levels, disrupting ovarian function, and increasing risk of chronic diseases of aging. This study demonstrates the additive negative effects of poor diet and psychosocial stress on ovarian physiology in mid-life and lays the groundwork for future investigations to uncover associated metabolic signatures of accelerated aging. The results also suggest that a Mediterranean diet may exert a protective influence against ovarian dysfunction and its pathologic sequelae.

